# Electronic Cigarette Use in 12 European Countries: Results From the TackSHS Survey

**DOI:** 10.2188/jea.JE20210329

**Published:** 2023-06-05

**Authors:** Silvano Gallus, Alessandra Lugo, Chiara Stival, Sonia Cerrai, Luke Clancy, Filippos T. Filippidis, Giuseppe Gorini, Maria José Lopez, Ángel López-Nicolás, Sabrina Molinaro, Anna Odone, Joan B. Soriano, Olena Tigova, Piet A. van den Brandt, Constantine I. Vardavas, Esteve Fernandez, the TackSHS Project Investigators

**Affiliations:** 1 Department of Environmental Health Sciences, Istituto di Ricerche Farmacologiche Mario Negri IRCCS, Milan, Italy; 2 Epidemiology and Health Research Lab, Institute of Clinical Physiology, National Research Council of Italy (IFC-CNR), Pisa, Italy; 3 TobaccoFree Research Institute Ireland, Dublin, Ireland; 4 Department of Primary Care and Public Health, Imperial College London, London, United Kingdom; 5 Oncologic Network, Prevention and Research Institute (ISPRO), Florence, Italy; 6 Agència de Salut Pública de Barcelona, Barcelona, Spain; 7 Departament de Ciències Experimentals i de la Salut (DCEXS), Universitat Pompeu Fabra, Barcelona, Spain; 8 Consortium for Biomedical Research in Epidemiology and Public Health (CIBERESP), Madrid, Spain; 9 Sant Pau Institute of Biomedical Research (IIB Sant Pau), Barcelona, Spain; 10 Polytechnic University of Cartagena (UPCT), Cartagena, Spain; 11 School of Medicine, University Vita-Salute San Raffaele, Milan, Italy; 12 Department of Public Health, Experimental and Forensic Medicine, University of Pavia, Pavia, Italy; 13 Hospital Universitario La Princesa (IISP), Madrid, Spain; 14 Consortium for Biomedical Research in Respiratory Diseases (CIBERES), Madrid, Spain; 15 Tobacco Control Research Group, Epidemiology and Public Health Research Programme, Institut d’Investigació Biomèdica de Bellvitge-IDIBELL, Barcelona, Spain; 16 Tobacco Control Unit, WHO collaborating center on tobacco control, Institut Català d’Oncologia-ICO, Barcelona, Spain; 17 School of Medicine and Health Sciences, Campus of Bellvitge, Universitat de Barcelona, Barcelona, Spain; 18 Maastricht University Medical Centre, GROW- School for Oncology and Developmental Biology, Department of Epidemiology, Maastricht, the Netherlands; 19 Maastricht University Medical Centre, CAPHRI- School for Public Health and Primary Care, Department of Epidemiology, Maastricht, the Netherlands; 20 Hellenic Cancer Society, Athens, Greece; 21 School of Medicine, University of Crete, Crete, Greece

**Keywords:** electronic cigarettes, ENDS, nicotine, cross-sectional study, Europe

## Abstract

**Background:**

Limited data on electronic cigarette prevalence, patterns, and settings of use are available from several European countries.

**Methods:**

Within the TackSHS project, a face-to-face survey was conducted in 2017–2018 in 12 European countries (Bulgaria, England, France, Germany, Greece, Ireland, Italy, Latvia, Poland, Portugal, Romania and Spain). Overall, 11,876 participants, representative of the population aged ⩾ 15 years in each country, provided information on electronic cigarette.

**Results:**

2.4% (95% confidence interval [CI], 2.2–2.7%) of the subjects (2.5% among men and 2.4% among women; 0.4% among never, 4.4% among current- and 6.5% among ex-smokers) reported current use of electronic cigarette, ranging from 0.6% in Spain to 7.2% in England. Of the 272 electronic cigarette users, 52.6% were dual users (ie, users of both electronic and conventional cigarettes) and 58.8% used liquids with nicotine. In all, 65.1% reported using electronic cigarette in at least one indoor setting where smoking is forbidden; in particular, at workplaces (34.9%) and bars and restaurants (41.5%). Multivariable logistic regression analysis showed that electronic cigarette use was lower among older individuals (*P* for trend <0.001) and higher among individuals with high level of education (*P* for trend = 0.040). Participants from countries with higher tobacco cigarette prices more frequently reported electronic cigarette use (odds ratio 3.62; 95% CI, 1.80–7.30).

**Conclusion:**

Considering the whole adult population of these 12 European countries, more than 8.3 million people use electronic cigarettes. The majority of users also smoked conventional cigarettes, used electronic cigarettes with nicotine, and consumed electronic cigarettes in smoke-free indoor areas.

## INTRODUCTION

Electronic cigarettes are electronic devices that heat a liquid to generate an inhalable vapor, which may contain nicotine.^1^^,^^2^ In Europe, after an initial rapid spread since 2010,^3^ the proportion of adult regular electronic cigarette users rose from 1.5% in 2014 to 1.8% in 2017, with large differences among countries, depending on the fiscal and regulatory ‘interventions’ or ‘climate’ these products are subjected to across Europe.^4^ An Eurobarometer survey conducted in summer 2020 showed that more than 1 in 10 subject has at least tried e-cigarette, 9% having tried this product only once or twice, 3% having used it in the past, and 2% using it currently.^5^

Public health experts hold different opinions on the role of electronic cigarettes in tobacco control. Public Health England and electronic cigarette advocates promote the substitution of electronic cigarettes for combustible cigarettes among all smokers, hinting at possible harm reduction.^6^ On the other hand, international organizations, including the World Health Organization (WHO) and the European Commission, raised some concerns with the use of electronic cigarettes.^7^^,^^8^ The WHO recently warned against electronic cigarette use as a smoking cessation tool, stating that it is “undoubtedly harmful” and that the majority of electronic cigarette users are dual users (who concurrently use conventional and electronic cigarettes).^7^ Several studies have shown that electronic cigarettes have harmful health effects.^9^^,^^10^ Moreover, a few studies reported that electronic cigarettes are commonly used in indoor sites where smoking is forbidden,^11^^–^^13^ suggesting that smokers might decide to use electronic cigarettes in smoke-free areas to circumvent smoking bans and be able to maintain their daily intake of nicotine.^11^ At least two systematic reviews showed that electronic cigarettes help smokers to quit in clinical and/or controlled settings.^14^^,^^15^ However, this has not been supported in studies of real-world use.^7^^,^^15^ Moreover, ex-smokers having quit through electronic cigarettes could become long-term electronic cigarette users, which may have adverse implications from a public health perspective.^7^^,^^16^^,^^17^

Furthermore, there are even more concerns for non-smokers. Electronic cigarettes are promoted also to never smokers, particularly young people,^18^ and former smokers who quit. There is increasing evidence that electronic cigarettes constitute a gateway towards nicotine addiction (or even smoking combustible cigarettes) rather than an effective tool for harm reduction.^1^^,^^18^^,^^19^ In addition, some studies of adults found that among electronic cigarette users, non-smokers starting (or ex-smokers re-starting) smoking after using electronic cigarettes outnumbered current smokers who stop smoking after using electronic cigarettes.^1^^,^^20^

Given the rapid change in the use of tobacco and related products in European countries,^21^ it is important to provide updated data on the prevalence and characteristics of electronic cigarette users and the patterns of use, particularly in Europe. Comparisons among countries are difficult, however, since data are mostly based on national surveys and are collected differently across countries.^1^^,^^22^^–^^24^ Even the comparability of previous Eurobarometer data with the most recent estimates is difficult due to changes in the methodology.^25^ Thus, updated information on electronic cigarette use in different countries is needed, with a standardized assessment tool. In Europe, the prevalence of electronic cigarette use differed across countries: the United Kingdom had the highest prevalence in 2014 and 2017 (3.6% and 4.7%); the lowest prevalence was observed in Malta (0.0%) in 2014 and in Italy and Bulgaria (0.2%) in 2017.^4^ However, the landscape for electronic cigarettes is changing constantly, particularly in Europe,^3^ meaning that the prevalence and patterns of use of electronic cigarettes need to be monitored. Using data from the “Tackling second-hand tobacco smoke and electronic cigarette emissions: exposure assessment, novel interventions, impact on lung diseases and economic burden in diverse European populations” (TackSHS) survey, we illustrate the patterns of use of electronic cigarettes with country comparisons in 12 European countries.^26^^–^^28^

## METHODS

Within the TackSHS project,^29^ in 2017–2018 a survey was conducted in 12 strategically selected European countries (Bulgaria, England, France, Germany, Greece, Ireland, Italy, Latvia, Poland, Portugal, Romania, and Spain), representing geographical, legislative, and cultural variations across the European Union (EU) and covering about 80% of the whole EU-28 population (at the time of the survey). The fieldwork was conducted by Doxa, the Italian branch of the Worldwide Independent Network/Gallup International Association, and its European partners.^28^

In each country, we surveyed a sample of around 1,000 individuals aged 15 years and older (in England ⩾ 16 years and in Ireland ⩾ 18 years), representative of the general population in terms of age, sex, area of residence and—in most countries—socioeconomic characteristics. The survey comprised 11,902 subjects, representative of 342 million inhabitants aged 15 years or older of the 12 selected countries. Sampling methods differed by country: in Bulgaria, Greece, Italy, Latvia, and Romania, a multi-stage sampling was used with participants randomly selected to be representative of their population in terms of sex, age, and geographic area (in Italy, also by socio-economic status [SES]); in Germany, Ireland, Poland, Portugal, and Spain, stratified random sampling was used, combining also quotas on sex and age (in Ireland also social class); in England, cluster sampling was used with quotas on age, sex, SES, region, and urban/rural dwelling; and in France, quotas on age, sex, region, and city size were used.^28^

Ad hoc trained interviewers conducted the survey with computer-assisted personal interviewing in all 12 countries. The testing fieldwork was conducted by DOXA on 1,059 participants in November 2016 in Italy. The fieldwork in the other 11 countries was conducted between June 2017 (in Romania) and October 2018 (in Latvia).

Approval for the study was obtained from a local Ethics Committee in each of the 12 countries. The interviewers informed all participants about details of the survey and all participants provided their consent. The study protocol is registered at ClinicalTrials.gov (NCT02928536).

Besides information on demographic (eg, sex and age) and socio-economic characteristics (eg, level of education and self-assessed household economic status relative to the country-specific population), a specific section of the questionnaire focused on electronic cigarettes. Participants were asked if they: i) had tried electronic cigarettes once or twice in their life; ii) had used them in the past but not over the last 30 days (past users); iii) used them occasionally (5 days or less in the previous 30 days); or iv) used them regularly (more than 5 days in the previous 30 days). Current electronic cigarette users were those reporting electronic cigarette use either occasionally or regularly. Past and current electronic cigarette users provided information on their approximate number of puffs per day, the type of electronic cigarette used (with or without nicotine), and the type of device used (rechargeable, disposable, or mods and variable voltage devices).

Users also provided information on electronic cigarette consumption (ie, number of puffs per day; separately for working and non-working days) in each of selected indoor areas, including at home, at work, in public transport, in private cars, and in all other indoor places (including restaurants and bars).

Electronic cigarette users were also asked if they had visited specific sites (indoors, indoor transport, and outdoors) in the previous 6 months and if they had used electronic cigarettes during their last visit to each site. Indoor sites (excluding smoking areas) included: i) a friend’s or relative’s home, ii) drinking establishments (such as bars), iii) eating establishments (restaurants), iv) disco/club/concerts in indoor arenas, v) cinema/theatres, vi) courses or classes in hobbies/sports, vii) public libraries/government offices, viii) indoor train stations or subway stops, ix) airports, and x) healthcare centers (eg, hospitals). Indoor transports included: i) cars/private vehicles together with at least one minor (aged <18 years), ii) cars/private vehicles without minors, iii) public transport (tram/bus/subway), iv) trains, and v) airplanes. Outdoor sites included: i) patios of restaurants or bars, ii) trams/bus/subway stops, iii) outdoor areas of hospitals, iv) outdoor areas of schools, v) parks, vi) children’s playgrounds, vi) stadiums/outdoor arenas, vii) beaches, viii) motorbikes/scooters, and ix) bicycles.

The questionnaire also included a section on smoking habits. Never smokers were defined as participants who had never smoked or had smoked less than 100 cigarettes in their lifetime. Ever smokers were the participants who reported to have smoked at least 100 cigarettes (including hand-rolled cigarettes) during their lifetime. Current smokers were the subjects who reported to be currently smoking at the time of the interview, while ex-smokers were those who had stopped smoking by the time they participated in the study.

The 12 countries were classified according to their gross domestic product (GDP) per capita^30^: <€25,000 (Latvia, Romania, Poland, Portugal, Greece, Bulgaria) and ≥€25,000 (England, France, Germany, Ireland, Italy, Spain); according to the score of 2016 version of the Tobacco Control Scale (TCS; the score attributed to each country increases with the strength of tobacco control policies up to a maximum of 100 points, indicating full implementation)^31^: ≤50 (Bulgaria, Germany, Greece, Latvia, Poland, Portugal) and >50 (Spain, France, Ireland, Italy, Romania, England); and according to the component with the greatest weight in the 2016 version of the TCS (ie, the component referring to the average price standardized by GDP per capita of cigarettes in the country, with a maximum score equal to 30, indicating the highest standardized cigarette price) as a proxy of the affordability of tobacco in each country: <16 (Germany, Italy, Latvia, Poland, Spain) and ≥16 (England, France, Ireland, Romania, Portugal, Greece, Bulgaria).^32^

### Statistical analysis

In each country, statistical weights were computed and applied to assure the same age, sex, and geographic area specific distribution of each country using as a standard the data from the corresponding National Institute of Statistics. Estimates for the entire sample were performed using “country weights,” combining individual weights with an additional weighting factor, each country contributing in proportion to its population aged 15 years or over, according to Eurostat 2018.^33^

To take into account the heterogeneity between the 12 countries, odds ratios (ORs) and their 95% confidence intervals (CIs) for current versus non-use of electronic cigarettes were calculated with multilevel logistic random-effects models. The study country effects were considered as random intercepts, whereas sex, age, level of education, and smoking status were considered as adjusting variables. Country weights were used in all logistic regression models. All the analyses were done with SAS 9.4 (SAS Institute; Cary, NC, USA).

## RESULTS

**Figure 1.**
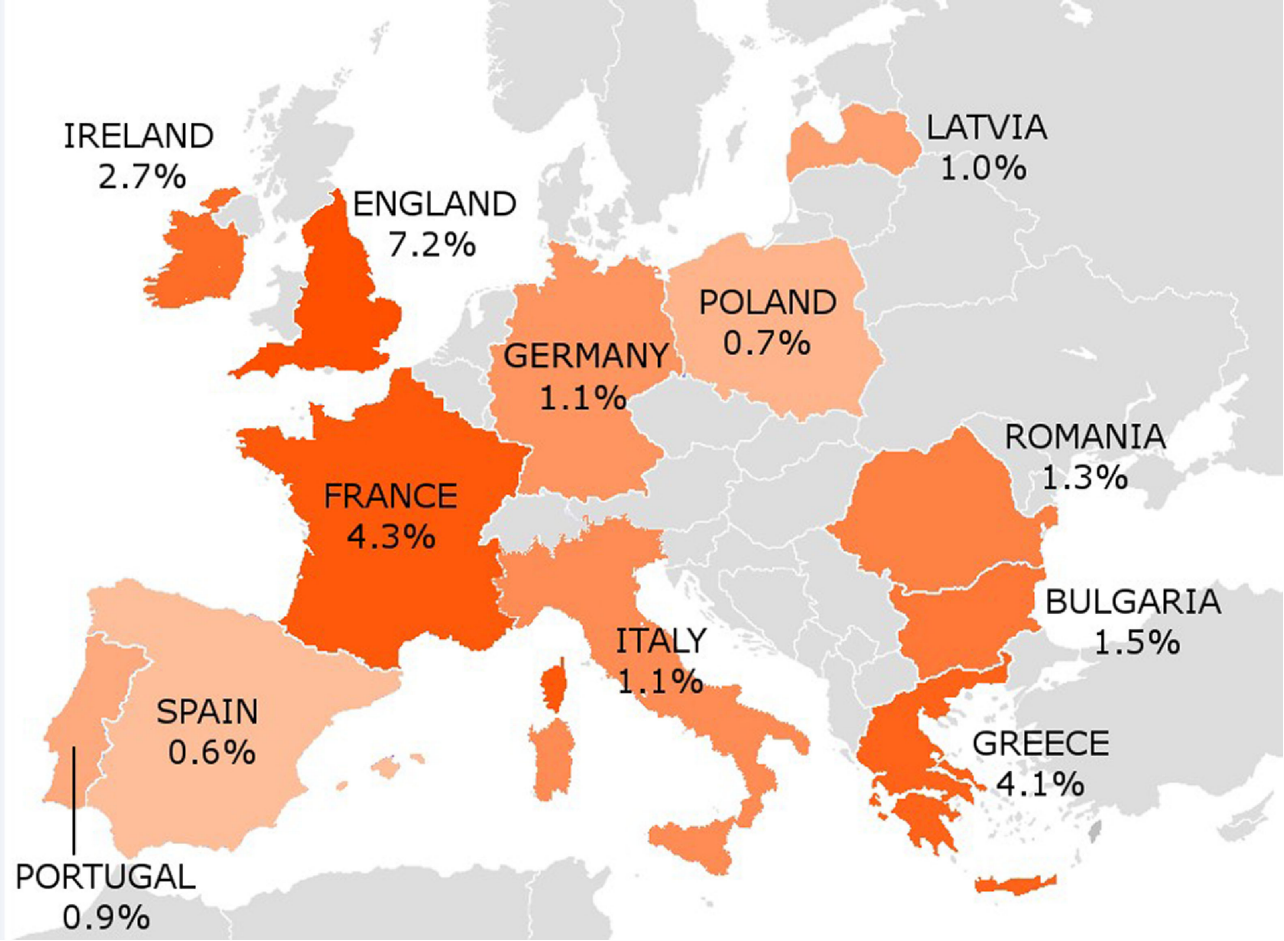
Country-specific prevalence (%)^a^ of electronic cigarette use among subjects aged ≥15 years in 12 selected European countries: TackSHS, 2017–2018. ^a^Country weights were applied, which combined individual weights with an additional weighting factor, each country contributing in proportion to its population aged 15 years or over (from Eurostat).^33^

Of the 11,902 participants, 26 did not provide information on electronic cigarette use. All the analyses are based on the remaining 11,876 (99.8%) participants. Among all participants, the prevalence of current electronic cigarette users was 2.4%, with the highest estimates in England (7.2%), France (4.3%), and Greece (4.1%) and the lowest in Spain (0.6%), Poland (0.7%), and Portugal (0.9%; Figure 1 and Table 1). The prevalence of electronic cigarette use was 2.5% in men and 2.4% in women. Overall, the prevalence of electronic cigarette use was 0.4% in never smokers, 4.4% in current smokers, and 6.5% in ex-smokers.

**Table 1.** Country-specific prevalence estimates of electronic cigarette use and corresponding 95% confidence intervals in 12 European countries among adult population aged ≥15 years: TackSHS, 2017–2018

Country	Number^a^	Never users (%; 95% CI)	Tried 1–2 times (%; 95% CI)	Past users (%; 95% CI)	Occasional users (%; 95% CI)	Regular users (%; 95% CI)	Current users (%; 95% CI)					

							Total	Sex		Smoking status		

								Men	Women	Never	Current	Former
Total^b^	11,876	87.0	8.1	2.4	0.7	1.7	2.4	2.5	2.4	0.4	4.4	6.5
		86.4–87.6	7.7–8.6	2.2–2.7	0.5–0.8	1.5–2.0	2.2–2.7	2.1–2.9	2.0–2.7	0.3–0.5	3.7–5.2	5.4–7.6
Bulgaria	1,050	81.9	13.3	3.4	0.7	0.8	1.5	2.0	1.0	0.4	3.0	1.1
		79.6–84.2	11.2–15.3	2.3–4.5	0.2–1.2	0.3–1.3	0.7–2.2	0.8–3.2	0.2–1.8	0.0–0.9	1.3–4.7	0.0–2.6
England	1,013	79.6	9.6	3.7	1.5	5.7	7.2	6.4	7.9	0.3	13.9	21.0
		77.1–82.1	7.8–11.4	2.5–4.8	0.7–2.2	4.3–7.2	5.6–8.8	4.1–8.6	5.6–10.1	0.0–1.2	9.1–18.7	15.4–26.6
France	1,018	79.7	11.4	4.6	1.2	3.1	4.3	3.9	4.7	0.8	7.6	7.8
		77.2–82.1	9.4–13.4	3.3–5.9	0.5–1.8	2.1–4.2	3.1–5.6	2.2–5.6	2.9–6.5	0.2–2.1	4.7–10.5	4.1–11.5
Germany	1,013	94.0	4.7	0.2	0.2	0.8	1.1	1.7	0.5	0.6	1.2	3.1
		92.5–95.5	3.4–6.0	0.0–0.5	0.0–0.6	0.3–1.4	0.4–1.7	0.5–2.8	0.0–1.1	0.0–1.2	0.0–2.6	0.1–6.1
Greece	1,000	74.2	14.2	7.5	1.3	2.8	4.1	4.4	3.8	0.0	8.6	4.7
		71.5–76.9	12.0–16.4	5.9–9.1	0.6–2.0	1.8–3.8	2.9–5.3	2.6–6.2	2.1–5.5	—	5.6–11.6	2.1–7.3
Ireland	941	86.8	9.1	1.4	0.7	2.1	2.7	2.7	2.7	0.6	5.6	8.1
		84.6–89.0	7.2–10.9	0.7–2.2	0.1–1.2	1.2–3.0	1.7–3.8	1.2–4.2	1.3–4.2	0.0–1.3	2.3–8.9	3.6–12.6
Italy	1,059	90.1	6.6	2.2	0.7	0.4	1.1	1.8	0.5	0.3	2.4	3.9
		88.3–91.9	5.1–8.1	1.3–3.1	0.2–1.2	0.0–0.8	0.5–1.7	0.6–2.9	0.0–1.0	0.0–0.7	0.3–4.6	0.4–7.3
Latvia	1,022	81.0	15.3	2.8	0.6	0.5	1.0	1.7	0.4	0.4	2.0	1.5
		78.6–83.4	13.0–17.5	1.8–3.8	0.1–1.0	0.0–0.9	0.4–1.6	0.5–2.8	0.0–1.0	0.0–0.9	0.4–3.6	0.0–3.3
Poland	724	90.2	6.6	2.6	0.1	0.6	0.7	1.1	0.3	0.3	1.3	1.4
		88.0–92.4	4.8–8.4	1.4–3.7	0.0–0.3	0.0–1.1	0.1–1.3	0.0–2.3	0.0–0.9	0.0–0.8	0.0–2.9	0.0–3.7
Portugal	1,000	89.3	9.7	0.1	0.3	0.6	0.9	1.3	0.6	0.0	1.4	2.7
		87.4–91.2	7.9–11.5	0.0–0.6	0.1–0.9	0.1–1.1	0.3–1.5	0.3–2.3	0.1–1.7	—	0.2–2.5	0.8–6.8
Romania	1,010	84.6	12.6	1.6	0.9	0.3	1.3	1.2	1.3	0.0	3.2	0.8
		82.3–86.8	10.6–14.7	0.8–2.4	0.3–1.5	0.0–0.7	0.6–1.9	0.2–2.1	0.3–2.3	—	1.4–5.1	0.0–2.0
Spain	1,026	91.1	6.5	1.7	0.3	0.3	0.6	0.7	0.5	0.0	1.5	0.6
		89.4–92.9	5.0–8.1	0.9–2.5	0.0–0.6	0.0–0.7	0.1–1.1	0.0–1.4	0.0–1.2	—	0.2–2.9	0.0–1.6

CI, confidence interval.

^a^Unweighted numbers.

^b^Country weights were applied, which combined individual weights with an additional weighting factor, each country contributing in proportion to its population aged 15 years or over (from Eurostat).^33^

Table 2 shows patterns of use for all 272 current electronic cigarette users. The median number of puffs per day was 50, and the proportion of dual users was 52.6% (95% CI, 46.6–58.5%). Overall, 58.8% (95% CI, 53.0–64.7%) of electronic cigarette users used liquids with nicotine. This proportion increased to 66.4% (95% CI, 58.7–74.2%) among dual users. The most commonly used type of device was rechargeable (82.4%; 95% CI, 77.8–86.9%), followed by mods and variable voltage devices (12.9%; 95% CI, 8.9–16.9%) and disposable devices (4.8%; 95% CI, 2.2–7.3%). In all, 80.2% (95% CI, 75.4–85.9%) of the electronic cigarette users reported using electronic cigarettes daily in indoor settings. The home was the place with the highest proportion of electronic cigarette use (73.5%; 95% CI, 68.3–78.8%).

**Table 2.** Patterns^a^ of current (occasional and regular) electronic cigarette use among 272 users in 12 European countries among adults aged ≥15 years: TackSHS, 2017–2018

Country	Number of current users	Number of puffs per day (median; IQR)	Dual Users (%)	Electronic cigarette with nicotine (%)	Type of electronic cigarette device (%)			Daily use in various indoor settings (%)					
	
					Rechargeable	Disposable	Mods	Home	Work	Public transports	Private cars	Other indoor settings	Any indoor settings
Total	272	50 (20–200)	52.6	58.8	82.4	4.8	12.9	73.5	34.9	8.8	27.2	34.6	80.2

Bulgaria	15	350 (100–1,000)	73.3	73.3	53.3	13.3	33.3	93.3	66.7	20.0	20.0	73.3	100.0
England	73	80 (30–100)	38.4	56.2	89.0	5.5	5.5	86.3	52.1	11.0	27.4	30.1	93.2
France	44	40 (20–70)	54.6	68.2	84.1	2.3	13.6	70.5	20.5	18.2	27.3	38.6	75.0
Germany	13	600 (30–1,000)	23.1	61.5	53.9	15.4	30.8	53.9	15.4	0.0	23.1	23.1	53.9
Greece	41	200 (15–1,000)	70.7	61.0	70.7	0.0	29.3	58.5	26.8	0.0	34.2	41.5	65.9
Ireland	25	40 (20–150)	40.0	44.0	88.0	8.0	4.0	76.0	20.0	8.0	28.0	12.0	84.0
Italy	14	35 (12–100)	50.0	64.3	92.9	0.0	7.1	78.6	42.9	7.1	28.6	35.7	78.6
Latvia	13	23 (20–50)	53.9	46.2	84.6	7.7	7.7	30.8	0.0	0.0	0.0	15.4	46.2
Poland	6	15 (5–50)	33.3	66.7	100.0	0.0	0.0	100.0	50.0	0.0	0.0	33.3	100.0
Portugal	9	99 (50–300)	55.6	55.6	88.9	0.0	11.1	77.8	44.4	0.0	44.4	33.3	88.9
Romania	11	20 (15–20)	90.9	54.6	90.9	9.1	0.0	81.8	45.5	9.1	27.3	45.5	90.9
Spain	8	25 (8–40)	87.5	50.0	100.0	0.0	0.0	62.5	25.0	12.5	50.0	50.0	75.0

IQR, interquartile range.

^a^All the estimates are unweighted.

**Figure 2.**
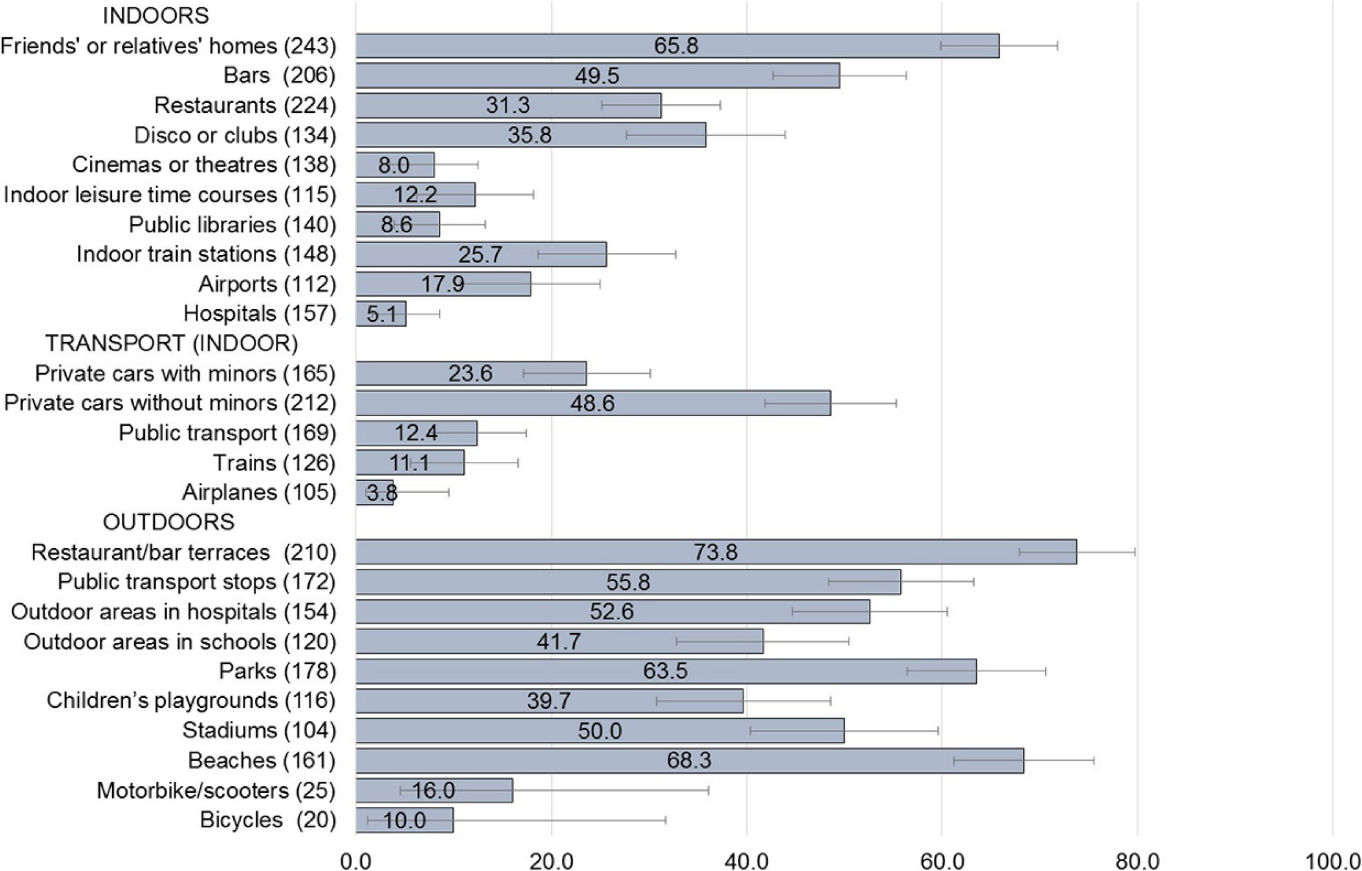
Use of electronic cigarettes in selected indoor and outdoor areas, among 272 users who visited the corresponding setting in the last 6 months in 12 European countries. Proportions (%) and corresponding 95% confidence intervals: TackSHS survey 2017–2018. For each setting, numbers in round brackets are number of the participants who visited the corresponding setting in the last 6 months and the percentages of participants who visited the setting in the last 6 months. CI, confidence interval.

Figure 2 and eTable 1 show the proportion of electronic cigarette use in selected indoor and outdoor sites the last time users visited each specific site (in the previous 6 months). Among electronic cigarette users having visited friends’ or relatives’ homes in the last 6 months, 65.8% reported having used electronic cigarettes on the last occasion; 49.5% of these users reported use in bars and 31.3% in restaurants. In the other indoor sites, electronic cigarette use ranged from 5.1% in hospitals to 35.8% in disco or clubs. Use in (indoor) transport ranged between 3.8% in airplanes and 48.6% in private cars without minors, with 23.6% using electronic cigarettes in private vehicles with minors present. For electronic cigarette users visiting outdoor settings over the last 6 months, those consuming electronic cigarettes ranged from 39.7% in children’s playgrounds to 73.8% on terraces of hospitality venues. Respectively, 16.0% and 10.0% of users consumed electronic cigarettes on motorbikes/scooters or bicycles.

Table 3 shows the proportion of users consuming electronic cigarettes in selected indoor sites where smoking is forbidden. In all, 65.1% (95% CI, 59.4–70.7%) reported using electronic cigarettes in one indoor setting, in particular workplaces (34.9%; 95% CI, 29.3–40.6%) and bars and restaurants (41.5%; 95% CI, 35.7–47.4%). The proportion of users consuming electronic cigarettes in places where smoking is forbidden ranged between 23.1% in Latvia and 93.3% in Bulgaria. Table 4 shows the ORs for current electronic cigarette users according to selected individual and country-specific characteristics. No statistically significant relationship was observed between sex and electronic cigarette use. Electronic cigarette use was lower in older individuals (*P* for trend <0.001) and higher in individuals with high level of education (*P* for trend = 0.040). There was no significant association between household economic status and electronic cigarette use. Compared to never smokers, the OR for current electronic cigarette use was 12.7 (95% CI, 8.3–19.4) for current smokers and 18.8 (95% CI, 12.2–29.0) for ex-smokers. There was also no significant relationship between GDP per capita or TCS score and electronic cigarette use, but participants from countries with higher cigarette prices more frequently reported electronic cigarette use compared to those from countries with lower prices (OR 3.62; 95% CI, 1.80–7.30).

**Table 3.** Distribution^a^ of 272 European current electronic cigarette users aged ≥15 years who used electronic cigarettes in workplaces and selected indoor public places where smoking is forbidden^b^: TackSHS, 2017–2018

Country	Number	Workplace (daily use)	Indoor public places where smoking is forbidden^b^ (%)					Workplace and any public places where smoking is forbidden

			Bars & Restaurants	Disco, cinemas, libraries^c^	Hospitals	Transport stations^d^	Public transport^e^	
Total^b^	272	34.9	41.5	22.1	2.9	15.8	9.9	65.1

Bulgaria	15	66.7	86.7	33.3	13.3	26.7	13.3	93.3
England	73	52.1	27.4	12.3	4.1	9.6	4.1	69.9
France	44	20.5	52.3	27.3	4.6	22.7	22.7	65.9
Germany	13	15.4	7.7	15.4	0.0	15.4	7.7	38.5
Greece	41	26.8	68.3	48.8	0.0	22.0	4.9	82.9
Ireland	25	20.0	32.0	12.0	4.0	12.0	12.0	40.0
Italy	14	42.9	50.0	35.7	0.0	28.6	21.4	71.4
Latvia	13	0.0	0.0	23.1	0.0	0.0	0.0	23.1
Poland	6	50.0	33.3	0.0	0.0	0.0	33.3	66.7
Portugal	9	44.4	22.2	11.1	0.0	11.1	0.0	55.6
Romania	11	45.5	54.6	0.0	0.0	9.1	0.0	72.7
Spain	8	25.0	37.5	0.0	0.0	25.0	12.5	50.0

^a^All the estimates are unweighted.

^b^Use of electronic cigarettes the last visit in each place is the last 6 months.

^c^This category includes disco or clubs, cinemas or theatres, indoor leisure time courses and public libraries.

^d^This category includes indoor train stations, subway stops and airports.

^e^This category includes public transport, trains and airplanes.

**Table 4.** Odds ratios for current electronic cigarette user versus non-users (never and former users combined) and corresponding 95% confidence intervals in the European population aged ≥15 years according to selected individual and country-specific characteristics^a^: TackSHS, 2017–2018

Characteristics	Number^b^	Number of Users^b^	%	OR (95% CI)^c^
Total	11,876	272	2.4	
Sex				
Female	6,253	131	2.4	1^d^
Male	5,623	141	2.5	0.91 (0.71–1.17)
Age group, years				
<25	1,441	40	2.3	1^d^
25–44	4,075	120	3.2	0.90 (0.61–1.35)
45–64	4,318	100	2.7	0.69 (0.46–1.03)
≥65	2,042	12	0.6	**0.22 (0.12–0.43)**
*P* for trend				**<0.001**
Level of education^e^				
Low	4,474	93	2.1	1^d^
Intermediate	4,162	98	2.2	0.93 (0.69–1.26)
High	3,237	81	3.2	**1.40 (1.03–1.89)**
*P* for trend				**0.040**
Self-reported household economic status^f^				
Lower than average	2,905	72	3.4	1^d^
Average	6,261	132	2.4	0.74 (0.54–1.00)
Higher than average	1,587	54	3.3	1.03 (0.69–1.52)
*P* for trend				0.777
Smoking status				
Never	6,502	24	0.4	1^d^
Current	3,326	143	4.4	**12.67 (8.29–19.35)**
Former	2,048	105	6.5	**18.83 (12.24–28.96)**
GDP per capita^g^				
≤25,000€	5,806	95	1.3	1^d^
>25,000€	6,070	177	2.8	2.25 (0.75–6.79)
TCS^h^				
≤50	5,809	97	1.2	1^d^
>50	6,067	175	3.2	2.10 (0.82–5.41)
TCS price component^i^				
<16	4,844	54	0.9	1^d^
≥16	7,032	218	4.5	**3.62 (1.80–7.30)**

CI, confidence interval; GDP, gross domestic product; OR, odds ratio; TCS, Tobacco Control Scale.

^a^Country weights combined individual weights with an additional weighting factor, with each country contributing in proportion to its population aged 15 years or over (from Eurostat).^33^

^b^Unweighted numbers.

^c^ORs and their 95% CIs were calculated using multilevel logistic random-effects models, to take into account the heterogeneity among the 12 European countries. The country effects were considered as random intercepts, and sex, age, level of education and smoking status as adjusting variables. Figures in bold type are significant at 0.05.

^d^Reference category.

^e^The sum does not add up to the total because of a few missing values.

^f^Self-assessment of household (family) economic status relative to the country-specific population. This variable is missing for all Germans, 79 Latvians and, 35 Romanians.

^g^GDP per capita ≤25,000€: BG, GR, LV, PO, PT, RO; >25,000€: ES, FR, GE, IE, IT, UK.

^h^TCS ≤50: BG, DE, GR, LV, PL, PT; TCS >50: ES, FR, IE, IT, RO, UK.

^i^TCS price component <16: DE, IT, LV, PL, ES; ≥16: BG, FR, GR, IE, PT, RO, UK.

eTable 2 shows the ORs for dual use among current electronic cigarette users according to selected characteristics. Dual users were less frequently males (OR 0.60; 95% CI, 0.36–1.00), older (*P* for trend <0.001) and with higher income (*P* for trend = 0.032).

## DISCUSSION

This face-to-face survey, based on a sample representing 80% of the whole EU-28 population in 2017–2018, shows that 2.4% of participants aged 15 years or more consumed electronic cigarettes in 12 European countries.

Electronic cigarette use still appears relatively limited in Europe, considering that 30% of current users are only occasional users (ie, using the product on 5 days or less in the previous month). However, compared to Eurobarometer data collected in 2014–2017,^4^ the prevalence of electronic cigarette use has increased in all countries, except for Germany and Poland, where it fell slightly.

The prevalence of electronic cigarette users differed substantially by country, from less than 1% in Spain, Poland, and Portugal to more than 7% in England. The wide spread of electronic cigarettes in the United Kingdom has been reported in several other studies^4^^,^^34^ and is probably explained by the early endorsement of electronic cigarettes by Public Health England followed by other United Kingdom institutions. This governmental agency has included electronic cigarettes among smoking cessation tools, recommending to improve access to these products for smokers in disadvantaged groups.^6^ However, in England, almost 40% of current electronic cigarette users are dual users (ie, also smoking conventional cigarettes). Considering all the 12 European countries combined, the proportion of dual electronic cigarette users exceeds 50%. This result, confirmed by most studies,^4^^,^^35^^,^^36^ once again highlights the lack of independent evidence on the effectiveness of these products as a population-level intervention to quit conventional tobacco use.^7^

One potential reason why people are dual users is to circumvent smoking bans. In agreement with other studies in Europe,^11^^–^^13^^,^^37^ we found that approximately two out of three users consumed electronic cigarettes in indoor smoke-free settings, particularly in workplaces, restaurants, and bars, where regulation on this novel products is still limited in Europe.^38^ It is increasingly evident that tobacco companies have been using electronic cigarettes and other novel tobacco products as a means to divert attention from effective tobacco control measures, thus possibly attenuating the effects of European smoking bans.

We confirmed findings from other studies showing that the majority of European electronic cigarette users use rechargeable devices.^11^^,^^39^ We found no indication of high use of disposable devices, although things might change rapidly, as has been recently shown among American youth.^40^

Moreover, most users—particularly current smokers—consume electronic cigarettes with nicotine.^39^^,^^41^ Half of non-smokers also use electronic cigarettes with nicotine, which increases the risk of starting tobacco smoking or relapsing.^1^^,^^42^^,^^43^

Current literature suggests that electronic cigarette use is equally prevalent among men and women.^36^ Accordingly, we did not find any significant difference in electronic cigarette use according to sex and in England current electronic cigarette users were even more frequently females. In agreement with current evidence from Europe, younger generations and people with a higher level of education are more likely to use electronic cigarettes.^44^ In our study, income did not appear to be a determinant of electronic cigarette use.

Besides England (7%), the prevalence of electronic cigarette users among adults was appreciable also in France (4%), Greece (4%), and Ireland (3%), all European countries where the price of cigarettes (standardized by per capita GDP) is relatively high.^32^ Thus, whereas we did not find any significant relationship between selected country-specific characteristics, including GDP per-capita and TCS, there was an inverse relationship between affordability of conventional cigarettes and electronic cigarette use. This suggests that, besides other factors—including public-health view of electronic cigarettes, the culture, and dynamics of the tobacco market—in countries where tobacco products are expensive, smokers are more likely to seek electronic cigarettes as (cheaper) alternatives.

The limitations of the present study include those inherent to cross-sectional studies with self-reported information, such as the impossibility to derive any causal inference from the results. We also note some differences in sampling methods in various study countries.^28^ The age ranges of the participants were also slightly different in some countries.^28^ However, differences were relatively minor and, consequently, estimates remain reasonably comparable. Moreover, we considered only EU countries. Another important aspect to take into account is that the small proportion of electronic cigarette users detected in the overall sample may affect the robustness of the estimates. Our findings should be interpreted with caution. In addition, our study provides a picture of electronic cigarette use at the investigated time (2017–2018). However, given the rapid evolution of the market of novel products, it is important to continue the monitoring of electronic cigarette use. The strengths of our survey include the representativeness of the adult population of 12 strategically selected European countries; the European Survey Tool (questionnaire),^28^ which was approved by a core of tobacco control experts and administered in the 12 countries sampled; the standardized definition of electronic cigarette use; and the use of face-to-face interviews. Finally, the large sample size enabled us to analyze endpoints with relatively low frequency, like the prevalence and patterns of electronic cigarette users. Generalized to the overall adult population, our data indicate that, in the 12 European countries included, 8.3 million people might be currently using electronic cigarettes. In most of the countries, current prevalence estimates have risen, with the highest prevalence in England. Most users are dual users, receiving nicotine from both electronic and conventional cigarettes. A large proportion of non-smokers use liquids with nicotine. Most users consume electronic cigarettes indoors and in places where smoking is forbidden. To discourage dual use, it is extremely important to regulate electronic cigarette use, banning this product at least in settings where smoking is already forbidden.

## SUPPLEMENTARY MATERIAL

eTable 1. Prevalence and 95% confidence intervals of electronic cigarette use among 272 users in selected indoor and outdoor sites in the last six months in 12 European countries: TackSHS survey 2017–2018

eTable 2. Odds ratios for dual users (current electronic cigarette user and conventional cigarette smokers) versus exclusive electronic cigarette users and corresponding 95% confidence intervals among 272 current European electronic cigarette users, according to selected characteristics^a^: TackSHS, 2017–2018

## References

[1] Liu X, Lugo A, Davoli E, . Electronic cigarettes in Italy: a tool for harm reduction or a gateway to smoking tobacco? Tob Control. 2020;29(2):148–152. 10.1136/tobaccocontrol-2018-054726 30659103

[2] World Health Organization. WHO Statement on ENDS/ENNDS. https://www.who.int/tobacco/communications/statements/eletronic-cigarettes-january-2017/en/; 2019 Access March 24, 2021.

[3] Filippidis FT, Laverty AA, Gerovasili V, . Two-year trends and predictors of e-cigarette use in 27 European Union member states. Tob Control. 2017;26(1):98–104. 10.1136/tobaccocontrol-2015-052771 27220621 PMC5256312

[4] Laverty AA, Filippidis FT, Vardavas CI. Patterns, trends and determinants of e-cigarette use in 28 European Union Member States 2014–2017. Prev Med. 2018;116:13–18. 10.1016/j.ypmed.2018.08.028 30144487

[5] Eurobarometer. Special Eurobarometer 506. Attitudes of Europeans towards tobacco and electronic cigarettes. Wave EB93.2 – Kantar. https://ec.europa.eu/commfrontoffice/publicopinion/index.cfm/ResultDoc/download/DocumentKy/91136. 2021 (Access March 22, 2021).

[6] McNeill A, Brose LS, Calder R, et al. Evidence review of e-cigarettes and heated tobacco products 2018. https://www.gov.uk/government/publications/e-cigarettes-and-heated-tobacco-products-evidence-review/evidence-review-of-e-cigarettes-and-heated-tobacco-products-2018-executive-summary#use-of-e-cigarettes-in-adults 2018 Access July 30, 2019.

[7] World Health Organization. WHO Report on the Global Tobacco Epidemic, 2019. Offer help to quit tobacco use. https://www.who.int/tobacco/global_report/en/; 2019 Access March 24, 2021.

[8] SCHEER. SHEER, Preliminary Opinion on electronic cigarettes. European Commission. https://ec.europa.eu/health/sites/health/files/scientific_committees/scheer/docs/scheer_o_017.pdf; 2020 Access March 24, 2021.

[9] Bhatta DN, Glantz SA. Association of e-cigarette use with respiratory disease among adults: a longitudinal analysis. Am J Prev Med. 2020;58(2):182–190. 10.1016/j.amepre.2019.07.028 31859175 PMC6981012

[10] Wills TA, Soneji SS, Choi K, . E-cigarette use and respiratory disorders: an integrative review of converging evidence from epidemiological and laboratory studies. Eur Respir J. 2021;57(1):1901815. 10.1183/13993003.01815-2019 33154031 PMC7817920

[11] Gallus S, Borroni E, Liu X, . Electronic cigarette use among Italian smokers: patterns, settings, and adverse events. Tumori. 2020;300891620915784. 10.1177/0300891620915784 32338200

[12] Shi Y, Cummins SE, Zhu SH. Use of electronic cigarettes in smoke-free environments. Tob Control. 2017;26(e1):e19–e22. 10.1136/tobaccocontrol-2016-053118 27609779 PMC5342954

[13] Matilla-Santander N, Fu M, Ballbè M, . Use of electronic cigarettes in public and private settings in Barcelona (Spain). Environ Res. 2017;158:685–690. 10.1016/j.envres.2017.07.019 28734255

[14] Hartmann-Boyce J, McRobbie H, Lindson N, . Electronic cigarettes for smoking cessation. Cochrane Database Syst Rev. 2020;10:CD010216. 10.1002/14651858.CD010216.pub4 33052602 PMC8094228

[15] Wang RJ, Bhadriraju S, Glantz SA. E-cigarette use and adult cigarette smoking cessation: a meta-analysis. Am J Public Health. 2021;111(2):230–246. 10.2105/AJPH.2020.305999 33351653 PMC7811087

[16] Pisinger C, Vestbo J. A new Cochrane review on electronic cigarettes for smoking cessation: should we change our practice? Eur Respir J. 2020;56(6):2004083. 10.1183/13993003.04083-2020 33361450

[17] Borrelli B, O’Connor GT. E-cigarettes to assist with smoking cessation. N Engl J Med. 2019;380(7):678–679. 10.1056/NEJMe1816406 30699299

[18] Conner M, Grogan S, Simms-Ellis R, . Do electronic cigarettes increase cigarette smoking in UK adolescents? Evidence from a 12-month prospective study. Tob Control. 2018;27(4):365–372. 10.1136/tobaccocontrol-2016-053539 28818839 PMC6047139

[19] Gallus S, Lugo A, Pacifici R, . E-cigarette awareness, use, and harm perceptions in Italy: a national representative survey. Nicotine Tob Res. 2014;16(12):1541–1548. 10.1093/ntr/ntu124 25082832

[20] Coleman B, Rostron B, Johnson SE, . Transitions in electronic cigarette use among adults in the Population Assessment of Tobacco and Health (PATH) Study, Waves 1 and 2 (2013–2015). Tob Control. 2019;28(1):50–59. 10.1136/tobaccocontrol-2017-054174 29695458 PMC6202279

[21] Gallus S, Borroni E, Odone A, . The role of novel (tobacco) products on tobacco control in Italy. Int J Environ Res Public Health. 2021;18(4):1895. 10.3390/ijerph18041895 33669394 PMC7920305

[22] Eichler M, Blettner M, Singer S. The use of e-cigarettes. Dtsch Arztebl Int. 2016;113(50):847–854. 10.3238/arztebl.2016.0847 28098063 PMC5273587

[23] Hedman L, Backman H, Stridsman C, . Association of electronic cigarette use with smoking habits, demographic factors, and respiratory symptoms. JAMA Netw Open. 2018;1(3):e180789. 10.1001/jamanetworkopen.2018.0789 30646032 PMC6324524

[24] Lidón-Moyano C, Martínez-Sánchez JM, Fu M, . [Prevalence and user profile of electronic cigarettes in Spain (2014)]. Gac Sanit. 2016;30(6):432–437. 10.1016/j.gaceta.2016.03.010 27198921

[25] Eurobarometer. Special Eurobarometer 458. Attitudes of Europeans towards tobacco and electronic cigarettes. Wave EB87.1–TNS opinion & social. https://op.europa.eu/en/publication-detail/-/publication/2f01a3d1-0af2-11e8-966a-01aa75ed71a1; 2017 Access March 22, 2021.

[26] Gallus S, Lugo A, Liu X, . Use and awareness of heated tobacco products in Europe. J Epidemiol. 2022;32(3):139–144. 10.2188/jea.JE20200248 33456019 PMC8824661

[27] Amalia B, Liu X, Lugo A, . Exposure to secondhand aerosol of electronic cigarettes in indoor settings in 12 European countries: data from the TackSHS survey. Tob Control. 2021;30(1):49–56. 10.1136/tobaccocontrol-2019-055376 32123139

[28] Gallus S, Lugo A, Liu X, . Who smokes in Europe? Data from 12 European countries in the TackSHS Survey (2017–2018). J Epidemiol. 2021;31(2):145–151. 10.2188/jea.JE20190344 32249267 PMC7813769

[29] Fernández E, López MJ, Gallus S, . Tackling second-hand exposure to tobacco smoke and aerosols of electronic cigarettes: the TackSHS project protocol. Gac Sanit. 2020;34(1):77–82. 10.1016/j.gaceta.2019.07.002 31558386

[30] World Bank. GDP per capita (current US$), World Bank national accounts data and OECD National Accounts data files. https://data.worldbank.org/indicator/NY.GDP.PCAP.CD?locations=EU; 2018 Access August 1, 2019.

[31] Feliu A, Fernández E, Baena A, . The Tobacco Control Scale as a research tool to measure country-level tobacco control policy implementation. Tob Induc Dis. 2020;18:91. 10.18332/tid/128318 33192223 PMC7656742

[32] Joossens L, Raw M. The Tobacco control scale 2016 in Europe. https://www.tobaccocontrolscale.org/TCS2016.pdf; 2017 Access March 21, 2021.

[33] European Commission. Eurostat. Database. https://ec.europa.eu/eurostat/data/database. (Access July 10, 2019).

[34] Beard E, West R, Michie S, . Association of prevalence of electronic cigarette use with smoking cessation and cigarette consumption in England: a time-series analysis between 2006 and 2017. Addiction. 2020;115(5):961–974. 10.1111/add.14851 31621131 PMC7187187

[35] Vardavas CI, Filippidis FT, Agaku IT. Determinants and prevalence of e-cigarette use throughout the European Union: a secondary analysis of 26,566 youth and adults from 27 Countries. Tob Control. 2015;24(5):442–448. 10.1136/tobaccocontrol-2013-051394 24935441

[36] Levy DT, Yuan Z, Li Y. The prevalence and characteristics of e-cigarette users in the U.S. Int J Environ Res Public Health. 2017;14(10):1200. 10.3390/ijerph14101200 29019917 PMC5664701

[37] Kiyohara K, Tabuchi T. Electronic cigarette use in restaurants and workplaces where combustible tobacco smoking is not allowed: an Internet survey in Japan. Tob Control. 2018;27(3):254–257. 10.1136/tobaccocontrol-2016-053581 28601841

[38] Amalia B, Fu M, Feliu A, . Regulation of electronic cigarette use in public and private areas in 48 countries within the WHO European Region: a survey to in-country informants. J Epidemiol. 2022;32(3):131–138. 10.2188/jea.JE20200332 33342937 PMC8824658

[39] Kyriakos CN, Filippidis FT, Hitchman S, . Characteristics and correlates of electronic cigarette product attributes and undesirable events during e-cigarette use in six countries of the EUREST-PLUS ITC Europe Surveys. Tob Induc Dis. 2018;16:A1. 10.18332/tid/93545 31516457 PMC6659560

[40] Wang TW, Gentzke AS, Neff LJ, . Disposable e-cigarette use among U.S. youth - an emerging public health challenge. N Engl J Med. 2021;384(16):1573–1576. 10.1056/NEJMc2033943 33725431

[41] Farsalinos KE, Poulas K, Voudris V, . Electronic cigarette use in the European Union: analysis of a representative sample of 27,460 Europeans from 28 countries. Addiction. 2016;111(11):2032–2040. 10.1111/add.13506 27338716

[42] Soneji S, Barrington-Trimis JL, Wills TA, . Association between initial use of e-cigarettes and subsequent cigarette smoking among adolescents and young adults: a systematic review and meta-analysis. JAMA Pediatr. 2017;171(8):788–797. 10.1001/jamapediatrics.2017.1488 28654986 PMC5656237

[43] Zhong J, Cao S, Gong W, . Electronic cigarettes use and intention to cigarette smoking among never-smoking adolescents and young adults: a meta-analysis. Int J Environ Res Public Health. 2016;13(5):465. 10.3390/ijerph13050465 27153077 PMC4881090

[44] Ooms GI, Bosdriesz JR, Portrait FR, . Sociodemographic differences in the use of electronic nicotine delivery systems in the European Union. Nicotine Tob Res. 2016;18(5):724–729. 10.1093/ntr/ntv215 26438649

