# Supplementary material for: Electronic Cigarette Use in 12 European Countries: Results From the TackSHS Survey
Source: J Epidemiol. 2023 Jun 5;33(6):276–84. doi: 10.2188/jea.JE20210329 (PMC10165220; doi:10.2188/jea.JE20210329)
Supplement: Supplementary file 1 [file je-33-276-s001.pdf]

eTable 1. Prevalence and 95% confidence intervals of electronic cigarette use among 272 users in selected indoor and outdoor sites in the last six months in 12 European countries: TackSHS survey 2017–2018

Indoor and outdoor sites	Not visited in the last 6 months (%; 95% CI)	Visited in the last 6 months	
		No electronic cigarette use (%; 95% CI)	Electronic cigarette use (%; 95% CI)
Indoors (excluding smoking areas)			
Friends' or relatives' homes	6.5 (3.5–9.5)	31.9 (26.3–37.6)	61.5 (55.6–67.5)
Bars	22.9 (17.8–27.9)	39.0 (33.1–44.8)	38.2 (32.4–44.0)
Restaurants	16.4 (12.0–20.9)	57.5 (51.5–63.4)	26.1 (20.9–31.4)
Disco or clubs	50.4 (44.4–56.3)	31.9 (26.3–37.4)	17.8 (13.2–22.3)
Cinemas or theatres	49.1 (43.1–55.0)	46.9 (40.9–52.8)	4.1 (1.7–6.4)
Indoor leisure time courses	57.1 (51.2–63.0)	37.7 (31.9–43.5)	5.2 (2.6–7.9)
Public libraries	47.8 (41.8–53.7)	47.8 (41.8–53.7)	4.5 (2.0–7.0)
Indoor train stations	44.6 (38.6–50.5)	41.2 (35.3–47.1)	14.2 (10.0–18.4)
Airports	58.4 (52.5–64.3)	34.2 (28.5–39.9)	7.4 (4.3–10.6)
Hospitals	41.6 (35.7–47.5)	55.4 (49.5–61.3)	3.0 (0.9–5.0)
Transport (Indoor)			
Private cars with minors	38.4 (32.6–44.3)	47.0 (41.0–53.0)	14.6 (10.3–18.8)
Private cars without minors	20.9 (16.0–25.8)	40.7 (34.8–46.6)	38.4 (32.6–44.3)
Public transport	37.4 (31.6–43.2)	54.8 (48.9–60.8)	7.8 (4.6–11.0)
Trains	53.3 (47.4–59.3)	41.5 (35.6–47.4)	5.2 (2.5–7.8)
Airplanes	61.1 (55.3–66.9)	37.4 (31.6–43.2)	1.5 (0.4–3.8)
Outdoors			
Restaurant/bar terraces	21.6 (16.7–26.6)	20.5 (15.7–25.4)	57.8 (51.9–63.8)
Public transport stops	36.1 (30.3–41.8)	28.3 (22.9–33.6)	35.7 (30.0–41.4)
Outdoor areas in hospitals	42.8 (36.8–48.7)	27.1 (21.8–32.5)	30.1 (24.6–35.6)
Outdoor areas in schools	55.6 (49.6–61.5)	25.9 (20.7–31.2)	18.5 (13.9–23.2)
Parks	33.8 (28.2–39.5)	24.2 (19.1–29.3)	42.0 (36.1–47.9)
Children's playgrounds	56.7 (50.8–62.7)	26.1 (20.9–31.4)	17.2 (12.7–21.7)
Stadiums	61.5 (55.7–67.3)	19.3 (14.6–24.0)	19.3 (14.6–24.0)
Beaches	40.2 (34.3–46.0)	19.0 (14.3–23.6)	40.9 (35.0–46.8)
Motorbike/scooters	78.6 (71.2–86.1)	18.0 (11.0–24.9)	3.4 (0.9–8.5)
Bicycles	83.6 (77.0–90.2)	14.8 (8.5–21.1)	1.6 (0.2–5.8)

CI, confidence interval.

eTable 2. Odds ratios for dual users (current electronic cigarette user and conventional cigarette smokers) versus exclusive electronic cigarette users and corresponding 95% confidence intervals among 272 current European electronic cigarette users, according to selected characteristics[a]: TackSHS, 2017-2018

Characteristics	Total no. of current electronic cigarette users[b]	Dual users (%)	OR (95% CI)[c]
Total	272	52.6	
Sex			
Female	131	56.5	1[d]
Male	141	48.9	**0.60 (0.36–1.00)**
Age group, years			
<25	40	77.5	1[d]
25–44	120	51.7	**0.28 (0.12–0.64)**
45–64	100	46.0	**0.20 (0.08–0.47)**
≥65	12	33.3	**0.13 (0.03–0.56)**
P for trend			**<0.001**
Level of education [e]			
Low	93	53.8	1[d]
Intermediate	98	50.0	0.82 (0.45–1.48)
High	81	54.3	1.05 (0.56–1.95)
P for trend			0.911
Self-reported household economic status[f]			
Lower than average	72	65.3	1[d]
Average	132	49.2	**0.46 (0.24–0.85)**
Higher than average	54	50.0	**0.45 (0.20–0.98)**
P for trend			**0.032**

CI, confidence intervals; OR, odds ratio.

[a] Country weights combined individual weights with an additional weighting factor, with each country contributing in proportion to its population aged 15 years or over (from Eurostat).[33]

[b] Raw sample size

[c] ORs and their 95% CIs were calculated using multiple logistic regression models after adjustment for sex, age group and level of education. Estimates in bold are statistically significant at 0.05 level.

[d] Reference category

[e] The sum does not add up to the total because of a few missing values.

[f] Self-assessment of household (family) economic status relative to the country-specific population. The sum does not add up to the total because of a few missing values.
